# Microwave Based Non-Destructive Testing for Detecting Cold Welding Defects in Thermal Fusion Welded High-Density Polyethylene Pipes

**DOI:** 10.3390/polym17152048

**Published:** 2025-07-27

**Authors:** Zhen Wang, Chaoming Zhu, Jinping Pan, Ran Huang, Lianjiang Tan

**Affiliations:** 1 School of Materials Science and Engineering, Shanghai Institute of Technology, Shanghai 201418, China; jywangzhenyyds@163.com; 2 Jiaxing Special Equipment Inspection Institute, Jiaxing 314051, China; 18357353391@163.com (C.Z.); jxlpjp@foxmail.com (J.P.); 3 College of Biomedical Engineering, & Yiwu Research Institute, & Zhuhai Fudan Innovation Institute, Fudan University, Shanghai 200433, China; 4 Center for Innovation and Entrepreneurship, Taizhou Institute of Zhejiang University, Taizhou 318000, China

**Keywords:** high-density polyethylene pipes, thermal fusion welds, cold welding defects, microwave non-destructive testing

## Abstract

High-density polyethylene (HDPE) pipes are widely used in urban natural gas pipeline systems due to their excellent mechanical and chemical properties. However, welding joints are critical weak points in these pipelines, and defects, such as cold welding—caused by reduced temperature or/and insufficient pressure—pose significant safety risks. Traditional non-destructive testing (NDT) methods face challenges in detecting cold welding defects due to the polymer’s complex structure and characteristics. This study presents a microwave-based NDT system for detecting cold welding defects in thermal fusion welds of HDPE pipes. The system uses a focusing antenna with a resonant cavity, connected to a vector network analyzer (VNA), to measure changes in microwave parameters caused by cold welding defects in thermal fusion welds. Experiments conducted on HDPE pipes welded at different temperatures demonstrated the system’s effectiveness in identifying areas with a lack of fusion. Mechanical and microstructural analyses, including tensile tests and scanning electron microscopy (SEM), confirmed that cold welding defects lead to reduced mechanical properties and lower material density. The proposed microwave NDT method offers a sensitive, efficient approach for detecting cold welds in HDPE pipelines, enhancing pipeline integrity and safety.

## 1. Introduction

High-density polyethylene (HDPE) pipes possess numerous excellent properties, such as high toughness, corrosion resistance, good weathering resistance, superior resistance to scratch and crack propagation, etc. [1,2,3,4,5]. Due to these qualities, HDPE pipes are widely used in the construction of urban natural gas pipelines. In pipeline systems, the joints between pipes are weak points [6,7]. Therefore, the safe service of HDPE pipe systems is closely related to the quality of welding joints. The main connection methods for HDPE pipes are thermal fusion welding and electro-fusion welding, with thermal fusion welding being more commonly used, especially for connecting pipes with diameters larger than 200 mm. Currently, the mainstream detection method for HDPE pipelines is ultrasonic non-destructive testing [8]. Ultrasonic testing can directly obtain information about the internal condition of materials and identify macro-defects [9]. However, polyethylene is a polymeric material that absorbs and dissipates much ultrasonic energy, and due to its complex structure, it enhances the scattering of sound waves, which significantly increases the difficulty of ultrasonic testing for HDPE pipeline weld joints [10,11]. As a result, detecting defects in HDPE pipelines using ultrasonic technology is quite challenging.

Microwave non-destructive testing is a non-contact detection technology that analyzes changes in the electromagnetic properties of microwaves as they propagate through an object to reveal its internal structure and properties [12,13]. When microwaves propagate through a material, they are affected by its electromagnetic properties, causing changes in parameters, such as amplitude, phase, and frequency. By measuring and analyzing these parameter changes, the internal structural characteristics of the object can be determined [14,15,16,17]. HDPE is a dielectric material with a low relative permittivity and weak absorption of microwaves, allowing microwaves to have strong penetration and reflection capabilities within it. Changes in the internal structure of HDPE cause phenomena, such as reflection, refraction, and scattering of the microwaves, which can lead to variations in microwave parameters that can be measured using certain instruments, like a vector network analyzer.

Cold welding is a typical process defect in joints of HDPE pipelines [18]. Inappropriate welding parameters (such as temperature, pressure, etc.) during welding are the main causes of cold welding defects. Unlike macroscopic defects, like holes, cracks, and inclusions, cold welds do not have obvious external characteristics and are easily overlooked. Furthermore, existing conventional non-destructive testing methods are challenging to apply for detecting cold welding defects. Cold welds pose significant safety threats to HDPE pipe systems because, at the cold weld joint, the polyethylene molecules at the interface of the two pipes do not fully diffuse and entangle, resulting in insufficient molecular penetration. Although the material connection is established, the connection strength is not fully developed, and the mechanical properties are lower than those of normally welded joints.

There have been studies on the microwave detection of HDPE pipes [19,20]. K-band circular waveguides as well as K-band and K_a_-band rectangular waveguides were employed to detect cracks and other defects on the outer surface of HDPE pipes. The results indicate that microwave detection techniques can effectively identify these defects, with the rectangular waveguide yielding the best performance [21]. Carrigan et al. designed a crawling robot capable of moving inside pipes while carrying a microwave reflectometer to scan HDPE pipes, successfully detecting internal defects as small as 1 mm in width and 1 mm in depth [22]. However, most of the existing studies on microwave detection focus on detecting macroscopic defects, like holes and cracks, and do not address process defects in HDPE pipe joints. A U.S. company has developed and commercialized microwave non-destructive testing (NDT) equipment for HDPE pipes [23], but the technical details remain strictly confidential.

To enable the detection of cold welding defects, a focusing antenna with a resonant cavity was used as the probe, which was connected to a vector network analyzer (VNA) to comprise a microwave detection system. The system converts the effects of the tested sample on the antenna radiation into changes in the resonant parameters of the cavity, offering high sensitivity. Our research focuses on detecting cold welding defects in thermal fusion welds of HDPE pipes, and the results demonstrate that our microwave detection system can effectively identify cold welding defects of varying degrees.

## 2. Experimental Methods

### 2.1. Materials

**Figure 1:**
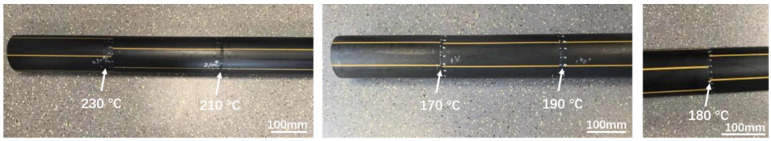
Thermal fusion welds of HDPE pipes jointed at different temperatures.

HDPE pipes (PE100, DN110) with a nominal diameter of 110 mm and a wall thickness of 10 mm were provided by Haining Chinaust Plastic Piping System Co., Ltd. (Jiaxing, China) The pipes contained thermal fusion welds formed at 230 °C, 210 °C, 190 °C, 180 °C and 170 °C, respectively, at the same welding pressure of 0.5 MPa, as shown in Figure 1. Furthermore, HDPE pipes with holes of 2 mm and 3 mm on the surface of pipe body were also used in this work.

### 2.2. Simulation

The structures of the resonant cavity, focusing antenna, and resonant probe were simulated by SolidWorks (v. 2023). The antenna simulation was conducted using the Ansys High-Frequency Structure Simulator (HFSS) software (v. 2023R1). The voltage standing wave ratio (VSWR) of the resonant probe was simulated using Python (v. 3.13). The electromagnetic simulation was performed within the 22–24 GHz frequency band. The computational model incorporates both the resonant cavity structure and focusing antenna, with geometries precisely matching the physical dimensions of the experimental system. The dielectric characteristics of HDPE were parameterized with a relative permittivity *ε_r_* of 2.58. Radiation boundary conditions were implemented at the simulation domain periphery to accurately model the unbounded electromagnetic environment surrounding the probe.

### 2.3. Microwave Test Systems and Principles

**Figure 2:**
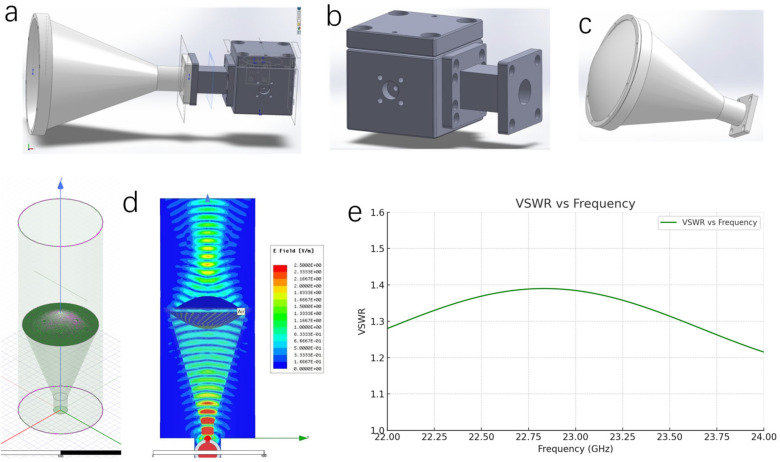
Simulation images of (**a**) the microwave resonant probe; (**b**) the resonator; (**c**) the focusing antenna. (**d**) Simulated diagrams of the resonating cavity coupled focusing antenna: radiation pattern (**left**); electric field distribution (**right**). (**e**) Simulated plot of VSWR versus frequency curve for the resonant probe.

As shown in Figure 2a–c, the microwave resonant probe primarily consists of a focusing antenna and a resonator. The resonator is a resonant cavity with two ports, namely a strongly coupled port and a weakly coupled port. The strongly coupled port of the resonant cavity is connected to the focusing antenna through a circular-to-rectangular waveguide transition, while the weakly coupled port is connected to a vector network analyzer (VNA, N5222B, Keysight Technologies, Santa Rosa, CA, USA) via a microwave cable. The resonant cavity operates in the transverse electric (TE) mode. The focusing antenna comprises a conical horn and a dielectric lens, with the lens being a double convex type positioned at the aperture of the conical horn. Figure 2d shows the simulated diagrams of the focusing antenna. The 3D radiation pattern represents the propagation of the electromagnetic field, with the intensity varying in concentric circles. The electric field distribution diagram illustrates the focused electric field within the antenna and the near-field distribution outside the antenna. The color map on the right indicates the intensity of the electric field in V/m (volts per meter). The resonant probe is designed to operate in the frequency range of 22–24 GHz. The VSWR of the probe was simulated, as shown in Figure 2e. The VSWR quantifies the degree of reflection in the transmission line, serving as an indicator of the probe’s efficiency in emitting microwave energy. The VSWR of 1.22 to 1.39 suggests low reflection loss and high emission efficiency.

**Figure 3:**
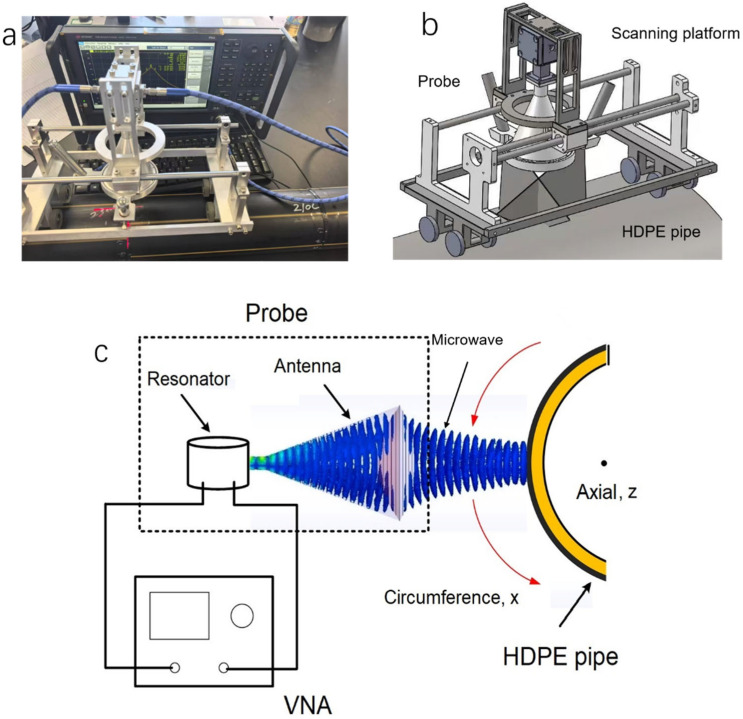
(**a**) Picture of the microwave testing system for inspecting thermal fusion welds of HDPE pipes. (**b**) Simulation model of the microwave resonant probe and the scanning platform. (**c**) Schematic illustration of inspecting an HDPE pipe using the microwave resonant probe.

When detecting HDPE pipes, the microwave resonant probe is mounted on a scanning platform, placed against the outer surface of the pipe, with the distance between the focusing antenna and the pipe surface (standoff distance) matching the antenna’s focal point (Figure 3a,b). The probe performs a two-dimensional scan along the circumferential (x-axis) and axial (z-axis) directions of the pipe (Figure 3c), while a laser positioner on the probe provides coordinate values of the probe’s position. In this study, thermal fusion welds of HDPE pipes were tested at a resonant frequency of 23.14 GHz. The penetration depth of microwaves at this frequency in HDPE exceeds 9 cm based on the skin effect [24], and, due to its high frequency, it provides sufficient resolution. A PTFE lens with a relative complex permittivity *ε_r_* of 2.1 and a focal length of 50 mm was chosen, resulting in a standoff distance of 50 mm during detection.

The two ports of the microwave resonant probe are connected to Port 1 and Port 2 of the VNA, respectively (Figure 3a). Microwaves resonate within the resonant cavity of the probe in a specific mode. During detection, the focusing antenna emits microwaves and concentrates the energy at the focal point. As the radiated microwaves encounter a sample, some of the energy is reflected back and interacts with the electromagnetic field within the resonant cavity, forming a stable resonance state. Once the materials or structures of the sample alter, the microwave reflection changes, causing disturbances to the resonance state and thereby changes in the cavity’s resonant parameters. The resonance curve revealing the resonance state in the cavity is obtained in real-time via the VNA. Herein, the resonance curve represents the variation in the magnitude of the S-parameter S_21_ as a function of frequency within the resonant frequency range. The changes in the resonant parameters caused by the sample are reflected in the variations of the resonance curve, including shifts in the magnitude and frequency of the resonance peak. These resonant parameters are highly sensitive to variations in the sample (e.g., various defects, impurities inclusion, material discontinuity), thereby providing the necessary conditions for detecting cold welding defects in thermal fusion welds of HDPE pipes.

### 2.4. Characterization

Tensile tests were performed using a CMT5504 electromechanical universal testing machine (MTS, Eden Prairie, MN, USA) at an elongation rate of 5 mm/min at room temperature. Scanning electron micrographs (SEMs) of the weld samples were recorded using a JSM-7401F field emission scanning electron microscope (JEOL, Tokyo, Japan). The weld samples were cut and sliced into small and thin pieces (5 × 5 × 2 mm) and were coated before SEM observation. A Ultrapyc 1200e Automatic Gas Pycnometer (Quantachrome Instruments, Boynton Beach, FL, USA) was used for measuring the true density and volume of the thermal fusion welds. Helium gas with a pressure of 12 psi was applied to the weld samples. The X-ray diffraction (XRD) pattern was recorded by a D/max-2200/PC X-ray diffractometer (Rigaku, Tokyo, Japan). The HDPE weld samples were cut into small pieces and powdered prior to XRD characterization.

## 3. Results and Discussion

### 3.1. The Microwave Inspection System for HDPE Pipes

**Figure 4:**
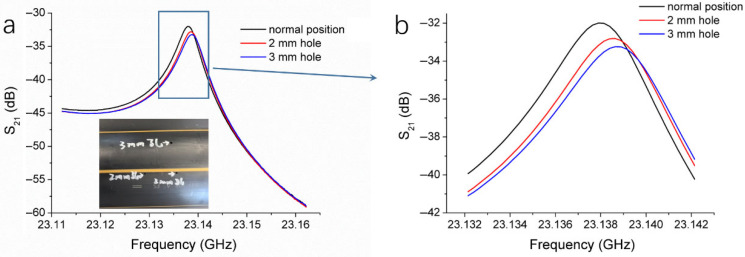
(**a**) Changes in the parameter S_21_ with frequency for HDPE pipes when the microwave resonant probe was located at different positions. The inset shows two HDPE pipes, with a 3 mm hole and two 2 mm holes on the surface, respectively. (**b**) The locally amplified S_21_ versus frequency curves within the box shown in (**a**).

To clarify the core principles of our microwave inspection system and its corresponding method, we chose voids as typical defects for testing, since the air has a very low permittivity. Holes with diameters of 2 mm and 3 mm, both with a depth of 10 mm, were drilled into the wall of an HDPE pipe, as shown in the inset of Figure 4. The HDPE pipe was scanned using the microwave resonant probe, with a constant standoff distance between the probe and the pipe surface throughout the entire scanning process. Three resonance curves were recorded, as follows: one when the probe was positioned at a normal (defect-free) location on the pipe, one at the 2 mm hole, and one at the 3 mm hole, as shown in Figure 4a. All resonance peaks were located within the frequency range of 23.135 to 23.140 GHz. Compared with the normal location, the resonance peak at the 2 mm hole exhibited a decrease in magnitude and a slight frequency shift. At the 3 mm hole, the peak magnitude was further reduced, and the peak position shifted towards higher frequencies. Figure 4b provides an enlarged view of the boxed region in Figure 4a, clearly showing the changes in the resonance curves caused by the presence of the 2 mm and 3 mm holes. The larger the hole, the more pronounced the changes, with greater decreases in resonance peak magnitude and larger frequency shifts.

As the probe moves along the surface of the HDPE pipe, the resonance curve remains virtually unchanged in defect-free areas. However, when the probe passes over a hole, the reflected microwave signal alters, disturbing the resonant state in the cavity and causing a noticeable change in the resonance curve compared to the defect-free condition. As the hole size increases, the disturbance to the resonant state becomes more pronounced. The permittivity and permeability of holes (air) are different from those of HDPE. This change in material influences the electromagnetic wave propagation characteristics and disturbs the electromagnetic field distribution, which in turn affects the reflected wave. For HDPE, variations in permeability are usually negligible. The reflection coefficient (*R*) at the interface between two dielectric media, which is a measure of how much of a microwave is reflected when it encounters the interface, can be calculated by a simplified model, as in the following Equation (1) [25]:

(1) $$R=\frac{\sqrt{\varepsilon_{2}}-\sqrt{\varepsilon_{1}}}{\sqrt{\varepsilon_{2}}+\sqrt{\varepsilon_{1}}}$$

where *ε*_1_ and *ε*_2_ are the relative permittivities of the two media. When air replaces HDPE in a hole, more microwaves are reflected at the hole position as the air has a permittivity lower than HDPE (*ε*_1_ ≠ *ε*_2_). The resonance state in the cavity is, thus, disturbed. The larger the size of the hole, the larger the area where reflection occurs. Therefore, the S_21_ magnitude of the resonance peak changes with the size of the holes.

In addition, changes in resonant frequency can be described by the following Equation (2) [26]:

(2) $$\frac{\omega_{2}-\omega_{1}}{\omega_{2}}=-\frac{\int_{V_{0}}\left(\Delta \varepsilon E_{2}\cdot E_{1}^{*}+\Delta \mu H_{2}\cdot H_{1}^{*}\right)dv}{\int_{V_{0}}\left(\varepsilon E_{2}\cdot E_{1}^{*}+\mu H_{2}\cdot H_{1}^{*}\right)dv}$$

where *ω*_1_ and *ω*_2_ are the angular frequency before and after the disturbance, respectively; *E*_1_ and *H*_1_ are electric and magnetic field distributions of the original system; *E*_2_ and *H*_2_ are electric and magnetic field distributions after the disturbance; Δ*ε* and Δ*µ* are the changes in the permittivity and permeability due to the disturbance, respectively. For the holes that replace the HDPE (Δ*µ* is usually negligible.), Δ*ε* < 0, because the permittivity of air is generally considered to be infinite. According to Equation (2), the resonance frequency increases as a hole replaces the HDPE, which explains the frequency shifts in Figure 4. These results demonstrate that our microwave non-destructive testing system is highly sensitive to changes in reflected microwave signal and capable of detecting material variations of HDPE pipes.

By connecting the ends of two HDPE pipes in a heated and molten state, a thermal fusion weld was formed upon cooling. Under molten conditions and applied pressure, the polyethylene molecular chains diffuse and are entangled, subsequently bonding together during the cooling process. Throughout the thermal fusion process, the molecular chain orientation and crystallinity of the pipe material may change, resulting in variations in the permittivity. Consequently, the response of the HDPE weld differs from that of the pipe body to microwave signals.

**Figure 5:**
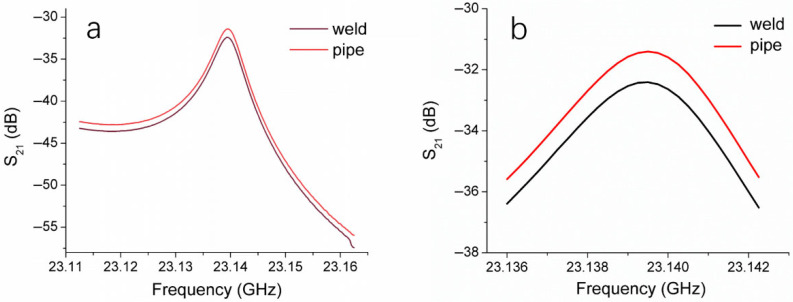
(**a**) Changes in S_21_ with frequency for the weld and the pipe body of an HDPE pipe. (**b**) The locally amplified S_21_ versus frequency curves in (**a**).

We employed the microwave resonant probe to test the thermal fusion weld of one HDPE pipe and a defect-free location on the pipe body, obtaining the resonance curves shown in Figure 5, with Figure 5b providing a magnified view of Figure 5a. The resonance curve of the thermal fusion weld differs from that of the pipe body, with the S_21_ magnitude at the resonance peak of the weld being lower, exhibiting a difference of 1.04 dB. This result indicates that the impact of fusion on the structural integrity of HDPE material can be detected by our microwave testing system, further confirming the feasibility of using a microwave resonant probe to assess the thermal fusion welds of HDPE pipes.

### 3.2. Detection of Cold Welding Defects

**Figure 6:**
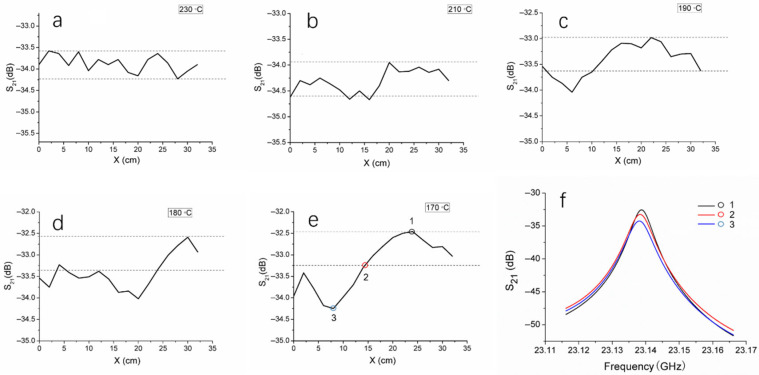
(**a**–**e**) Changes in S_21_ recorded during the process of microwave scanning around the outside surface of HDPE welds fused at different temperatures. The x-axis denotes the scanning distance of the probe around the welds. (**f**) The S_21_ versus frequency curves at the three points denoted in (**e**). For each plot in (**a**–**e**), the curve was obtained by recording the peak values of the S_21_ versus frequency curves at each point around the outside surface of HDPE welds.

To investigate the ability of the microwave system in identifying cold welding defects in thermal fusion welds of HDPE pipes, HDPE pipes were fused at five different temperatures, namely 170 °C, 180 °C, 190 °C, 210 °C, and 230 °C, producing five thermal fusion welds. The standard temperature range for thermal fusion of HDPE pipe is 210 °C to 230 °C, and temperatures below 210 °C may result in cold welding defects. The microwave resonant probe was positioned at the center of the weld and a 360° circumferential scan was conducted around the pipe, recording the S_21_ magnitude corresponding to the resonant peak at each position. The circumferential scanning distance was plotted on the x-axis, and the S_21_ magnitude on the y-axis, resulting in the scanning results of the five welds as shown in Figure 6.

At various positions along the circumference, the S_21_ magnitude fluctuations of the 230 °C weld were relatively small, with a difference of 0.66 dB between the maximum and minimum values, represented by the distance between the two dashed lines in Figure 6a. The temperature of 230 °C is the standard thermal fusion temperature, producing a densely structured fusion joint with a relatively uniform fusion surface. The S_21_ magnitude fluctuations for the 210 °C weld were similar to those of the 230 °C joint (Figure 6b), with a difference of 0.69 dB between the maximum and minimum values, which is very close to the 0.66 dB. When the fusion temperature is below 210 °C, cold welding is highly likely to occur. As shown in Figure 6c–e, the S_21_ magnitude fluctuations of the welds formed at 190 °C, 180 °C, and 170 °C are larger than those at 230 °C and 210 °C, and the fluctuation amplitude increases as the temperature decreases. In each plot, two dashed lines spaced 0.66 dB apart were added, with the upper dashed line positioned at the maximum S_21_ magnitude. It is reasonable to infer that when the S_21_ magnitude curve is between the two dashed lines, the fluctuations are acceptable, and the corresponding fusion area of the weld is defect-free; when the S_21_ magnitude curve falls outside the two dashed lines, a cold welding defect is present in the corresponding area. For the weld fused at 190 °C, the S_21_ magnitude falls outside the two dashed lines in the 0–10 cm region, indicating a lack of fusion in this region (Figure 6c). For the 180 °C weld, most of the 0–22 cm region shows the S_21_ magnitude outside the dashed lines, indicating a lack of fusion in this area (Figure 6d). The lack of fusion area in this weld is larger than that of the 190 °C weld. Additionally, the difference between the maximum and minimum S_21_ magnitudes for the 180 °C weld is 1.45 dB, while for the 190 °C weld, the difference is 1.07 dB, indicating that the degree of lack of fusion in the 180 °C weld is greater than that in the 190 °C weld. For the 170 °C weld, though the lack of fusion area (0–15 cm) is smaller than that of the 180 °C weld, the difference between the maximum and minimum S_21_ magnitudes is 1.79 dB (Figure 6e), demonstrating a more severe lack of fusion compared to the 180 °C weld.

Figure 6f shows the corresponding resonance curves at three points on the S_21_ magnitude curve in Figure 6e. Compared with the points located between the two dashed lines, which represent defect-free areas, the resonance curve peaks at points within the lack of fusion area are significantly lower. From the above results, it is evident that the S_21_ magnitude at defect locations is lower than at defect-free locations. The material structure at the lack of fusion areas is less compact, which may lead to a decrease in the permittivity. According to Equation (1), when microwaves pass through these areas, their reflection changes, and the resonant state within the cavity is disturbed, causing a change in the S_21_ magnitude. However, the resonance frequency seems unaffected by the lack of fusion. All these factors suggest that using a resonant probe to perform circumferential scanning along the center of weld can detect the presence and location of lack of fusion, thereby identifying cold weld and assessing their severity. Thermal fusion welds formed at temperatures below the standard fusion temperature will give rise to cold welding defects. As the fusion temperature decreases, the incidence and severity of defects increase.

**Figure 7:**
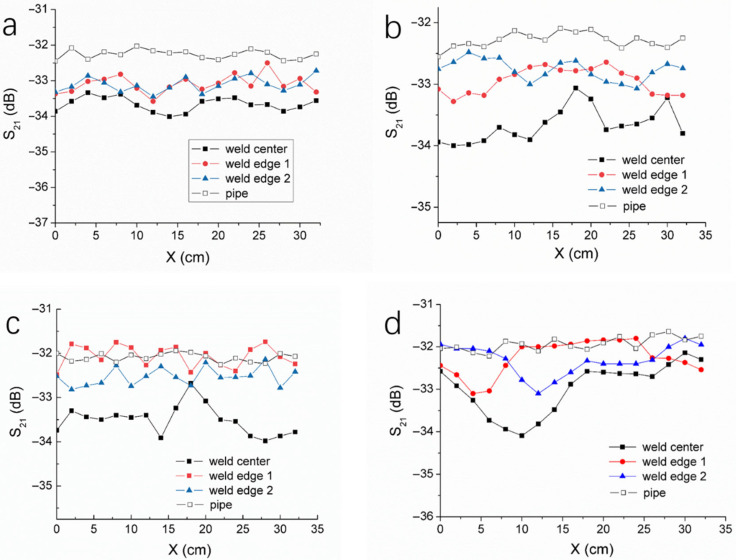
S_21_ versus circumferential distance for the thermal fusion welds formed at (**a**) 230 °C, (**b**) 190 °C, (**c**) 180 °C, and (**d**) 170 °C. For each weld, the circumferential scanning was performed with the probe against the weld center and two weld edges, respectively.

The thermal fusion weld of HDPE pipes has a width, and the heating and pressure conditions during welding differ between the center and the edges of the weld, leading to structural variations in the material. Microwave tests were conducted for the weld samples formed at 230 °C, 190 °C, 180 °C, and 170 °C, respectively. The microwave resonant probe was positioned at the center and the edges of each weld, performing a 360° circumferential scan. The results are shown in Figure 7, where the S_21_ curves obtained from the 360° circumferential scan of the pipe body are also shown. For the 230 °C weld, the S_21_ curve of the weld center and the two S_21_ curves of the weld edges exhibit relatively small fluctuations around the circumference, with the S_21_ magnitude at the weld center being smaller than those at the edges. Compared to the weld center and edges, the pipe body’s S_21_ magnitude is larger and shows smaller fluctuations. These results clearly demonstrate that significant microstructural differences exist between the weld zone and the base pipe material, and there are also structural differences between the center and the edges of the weld. This is primarily because, during the thermal fusion process, the PE molecular chains undergo thermal motion and entanglement under the influence of both temperature and pressure, followed by recrystallization during the cooling process, leading to changes in the material structure. The temperature and stress at the edges of the weld are different from those at the center, resulting in corresponding structural differences.

For the welds formed at 190 °C, 180 °C, and 170 °C, the magnitude of S_21_ at the weld center is also smaller than that at the weld edge and that of the pipe body. Additionally, the fluctuation in the S_21_ curves at the weld center is significantly greater than those at the weld edges, indicating that the edges of the weld are less affected by lack of fusion. This also suggests that, for better detection of cold welding defects, the probe should be positioned at the weld center and that a circumferential scan should be conducted along the weld seam. Compared with the welds formed at 190 °C and 180 °C, the three S_21_ curves of the weld formed at 170 °C exhibit a larger variation, further validating the capability of our microwave inspection method for detecting cold welds.

### 3.3. Microstructure and Mechanical Performance of Cold Welds

**Figure 8:**
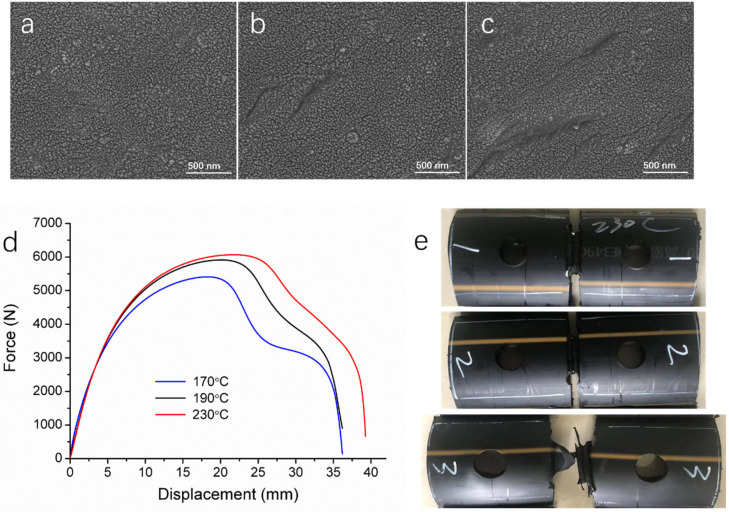
SEM images of sliced HDPE weld samples fused at (**a**) 230 °C, (**b**) 190 °C, and (**c**) 170 °C. (**d**) Tensile testing results of the welds fused at different temperatures. (**e**) Pictures of the weld samples after tensile testing. Welding temperature: 230 °C, 190 °C, and 170 °C from top to bottom, respectively.

As mentioned above, the lower temperature during thermal fusion hinders the diffusion, aggregation, and entanglement of polyethylene molecular chains, leading to alterations in the material structure and crystallinity of the weld compared to welds formed under normal conditions. This is the underlying cause of cold welding defects. To demonstrate the correlation between the material microstructure and the microwave detection, we conducted sampling analysis for cold welds. The microscopic morphology of welds formed at different temperatures was observed using SEM. As shown in Figure 8a, the microstructure of welds formed at the normal fusion temperature (230 °C) is uniform and dense, with no apparent defects. In contrast, the microstructures of welds formed at 190 °C and 170 °C exhibit grooves and microcracks, with the latter (170 °C) showing more pronounced defects (Figure 8b,c).

To further examine the differences in the structural compactness of normal welds and cold welds, true density analysis was adopted for the thermal fusion welds formed at various temperatures. True density refers to the actual mass per unit volume of a material in a fully dense state, excluding internal pores or voids. Cold welds typically result in localized lack of fusion within the weld, leading to less compact material in these areas, which may consequently exhibit lower true density. For true density measurements, small material samples cut from the welds were placed in appropriately sized cells. The results for welds formed at different temperatures, as well as for the pipe body, are presented in Table 1. The average true density values of welds fused at 230 °C and 210 °C were 0.9404 g/cm^3^ and 0.9397 g/cm^3^, respectively. These values are lower than the true density of the pipe body, 0.9530 g/cm^3^, indicating that thermal fusion welds formed at standard temperatures have a less compact microstructure compared to the pipe body. However, for welds fused at 190 °C, 180 °C, and 170 °C, the true density was even smaller, decreasing progressively with lower temperatures. This trend suggests that welds formed at lower temperatures possess less dense structures with more microvoids, as evidenced by their reduced true density values.

HDPE is a highly crystalline material. When the pipes are connected by thermal fusion, the PE crystals in the heating zone are melted and then recrystallize during the formation of welds. This process affects the crystallinity of the PE in the welds. The materials in the thermal fusion welds were sampled, powdered and characterized by XRD, the results of which are listed in Table 1. Compared with the pipe body (88.4%), the crystallinity of the welds formed at 230 °C and 210 °C is lower (84.2%), which is ascribed to the thermal fusion processing. As the welding temperature decreases, the crystallinity decreases. The crystallinity of the weld formed at 170 °C is 78.7%, 9.7% lower than that of the normal welds. The crystallinity and true density of thermal fusion welds demonstrate the structural differences between normal welds and cold welds, based on which, microwave non-destructive testing is able to identify the cold welding defects.

Since the presence of cold welding defects compromises the mechanical performance of thermal fusion welds, tensile tests were conducted on three weld samples, with the results shown in Figure 8d,e and Table 2. The weld formed at 230 °C exhibits the highest tensile strength, yield strength, and elongation at break. However, as the welding temperature decreases, the tensile mechanical properties deteriorate. The weld formed at 190 °C has a yield strength comparable to that of the weld formed at 230 °C but exhibits lower tensile strength and significantly reduced elongation at break. Meanwhile, the weld formed at 170 °C demonstrates even lower tensile strength and yield strength than the 190 °C weld, though it shows a similar elongation at break. The area under the tensile curve, which represents tensile work and reflects material toughness, is smaller for the two cold welds compared to the normal weld. This indicates that cold welds are likely more brittle and exhibit reduced toughness. The tensile curves and the post-fracture images of the welds (Figure 8e) reveal that all three welds undergo ductile fracture; however, the weld formed at 170 °C appears more brittle.

These findings underscore the adverse impact of cold welding defects on the mechanical performance of thermal fusion welds. Lower crystallinity decreases tensile strength in most cases. Lower density typically correlates with fewer voids and less porosity, which directly contributes to reduced mechanical properties. Therefore, accurate and efficient identification of cold welding defects using our microwave testing method is critical for ensuring the structural integrity and operational safety of HDPE pipelines.

## 4. Conclusions

This study demonstrates the effectiveness of a microwave-based NDT system for detecting cold welding defects in HDPE pipes. The microwave system, utilizing a focusing antenna and resonant cavity, was capable of identifying cold welds by detecting changes in resonance parameters. Experimental results showed that welds formed at temperatures lower than 210 °C exhibited significant variations in microwave signals, correlating with areas of insufficient fusion, a hallmark of cold welding defects. SEM observation and mechanical testing confirmed that these defects lead to more grooves and microcracks, thus reducing the tensile strength and toughness of the welded joints and compromising the structural integrity of the HDPE pipes. The microwave NDT method proves to be a highly sensitive and efficient approach for detecting cold welding defects where traditional NDT methods fall short. This technology can greatly enhance the safety and reliability of HDPE pipelines, making it a valuable tool for industries reliant on the integrity of polymer-based piping systems.

## Figures and Tables

**Table 1:** True density and crystallinity of pipe body and thermal fusion welds formed at different temperatures (n = 5).

Sample	True Density (g/cm^3^)	Crystallinity (%)
Pipe	0.9530 ± 0.0045	88.4 ± 2.0
230 °C	0.9404 ± 0.0033	84.2 ± 1.7
210 °C	0.9397 ± 0.0031	84.2 ± 2.1
190 °C	0.9313 ± 0.0037	82.1 ± 2.8
180 °C	0.9169 ± 0.0026	80.3 ± 2.5
170 °C	0.9085 ± 0.0038	77.6 ± 2.9

**Table 2:** Results of tensile testing for thermal fusion welds formed at different temperatures (n = 5).

Sample	Tensile Strength (MPa)	Yield Strength (MPa)	Elongation at Break (%)
230 °C	23.39 ± 0.59	21.26 ± 0.68	39.26 ± 1.16
190 °C	22.19 ± 0.43	21.02 ± 0.54	36.33 ± 0.91
170 °C	20.99 ± 0.49	19.60 ± 0.73	36.19 ± 1.04

## Data Availability

The original contributions presented in this study are included in the article. Further inquiries can be directed to the corresponding authors.
